# Isolated Solitary Lung Nodule in a Patient With Idiopathic Pulmonary Fibrosis and Concomitant Prostate Cancer: A Challenging Diagnosis

**DOI:** 10.7759/cureus.14218

**Published:** 2021-03-31

**Authors:** Mohamad Tarabaih, Jad A Degheili, Mouhamad Nasser

**Affiliations:** 1 Department of Oncology, Hôpital Lyon Sud, Institut de Carcinologie des Hospices Civils de Lyon (ICHCL), Lyon, FRA; 2 Division of Pediatric Urology, Department of Surgery, Children's Hospital of Eastern Ontario, University of Ottawa, Ottawa, CAN; 3 Division of Urology, Department of Surgery, American University of Beirut Medical Center, Beirut, LBN; 4 Department of Respiratory Medicine, National Coordinating Reference Centre for Rare Pulmonary Diseases, Louis Pradel Hospital, University Hospital of Lyon, Lyon, FRA

**Keywords:** isolated lung nodule, prostate cancer, prostate-specific antigen, idiopathic pulmonary fibrosis

## Abstract

Prostate cancer is the most commonly diagnosed malignancy and the second most common cause of death in men after lung cancer. Isolated pulmonary metastasis from prostate cancer, without bone or lymph node involvement, is rare and accounts for less than 1% of cases. The diagnosis of solitary lung metastasis is even more challenging in patients with concomitant pulmonary disease and often mandates tissue biopsy from the lung nodule. We herein present a case of an elderly man with idiopathic pulmonary fibrosis who presented with a solitary lung nodule three years after a laparoscopic radical prostatectomy for localized prostate cancer. Initially thought as a primary lung lesion secondary to his pulmonary fibrosis, further workup and ultimately a lung segmentectomy proved a metastatic prostatic adenocarcinoma. The serum prostatic specific antigen dropped to nadir following resection, and he remained stable six months later.

## Introduction

Idiopathic pulmonary fibrosis (IPF) is a chronic fibrotic lung disease characterized by a progressive and irreversible decline in lung function leading to increased dyspnea and impaired quality of life [1]. Male sex, smoking history, and increased age are all risk factors for developing IPF [2]. Furthermore, IPF is a well-known risk factor for lung cancer, with a hazard ratio of 2.77 [3].

Prostate cancer (PCa), which is the most common cancer diagnosed in men worldwide [4], can often metastasize with an elevated rate in patients with increased T-stage, histopathological Gleason score, and prostate-specific antigen (PSA) [5]. Emergence of new radiological techniques such as positron emission tomography (PET) with prostate-specific membrane antigen (PSMA), using the gadolinium-68 radiotracer, has widely changed the definition of metastatic disease [6]. Serum PSA is used to guide physicians for choosing the correct radiological test among others. For example, a cut-off PSA level of 500 ng/mL exhibits an average post-test probability of 76% for metastasis, 94% in patients with higher disease stage of T3-T4, and 50% to 63% in patients with lower stages, i.e., T1 and T2 disease [5]. In general, site-specific metastasis affects survival outcomes [7].

Bone is the most common site of metastasis (in 84% of cases) followed by distant lymph nodes (10.6%), liver (10.2%), and, finally, the thorax in 9.1% of cases [8]. Lung metastasis develops in nearly 27% of patients, but mostly in association with other site metastases [9]. Isolated lung metastasis has been described with an appreciable diagnostic challenge. IPF carries an increased risk of lung cancer (4.4% to 13%), and solitary lung nodules are commonly considered suspicious in patients with IPF [10,11]. The concomitant presence of PCa in such patients adds further challenge to physicians in determining the exact nature of a given nodule. Herein, we report a case of a 70-year-old man with IPF, diagnosed with a solitary lung nodule and resected, assumingly it was a malignant lung nodule given the poor uptake on 68-Ga PET/CT PSMA scans; but surprisingly, the pathology came as a metastatic adenocarcinoma, of prostatic origin, three years after his initial diagnosis with localized PCa.

## Case presentation

A 70-year-old male, a former smoker, was diagnosed previously with IPF based on the characteristic usual interstitial pneumonia pattern, as visualized on previous CT chests, with negative auto-immune serology workup. He then presented, in early 2017, with lower urinary tract symptoms and an elevated serum PSA of 11.05 ng/mL. A trans-rectal ultrasound-guided biopsy revealed a Gleason 7(4+3) prostate adenocarcinoma in both lobes, with a negative metastatic workup. The patient was thereafter diagnosed with localized PCa. He underwent a laparoscopic radical prostatectomy with pelvic lymph nodes dissection, with a final pathology of pT2c Gleason 7(4+3) N0R0. He refused any adjuvant hormonal therapy. His post-operative PSA nadir level dropped to 0.12 ng/mL.

**Figure 1:**
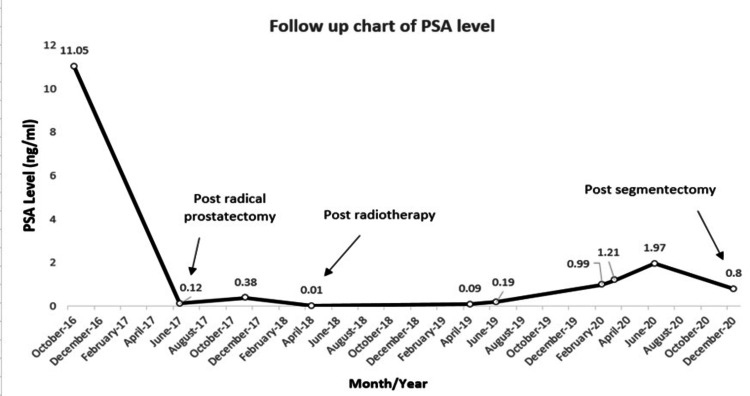
Trending of PSA levels during the course of our patient’s treatment: from the initial time of diagnosis until the last follow-up available. PSA, prostate-specific antigen

Upon routine follow-up, his serum PSA level increased to 0.38 ng/mL by end of 2017, with hypermetabolic external iliac and right inguinal metastatic adenopathies detected on C-11 choline PET/CT scan. The patient received an external beam radiation therapy to the whole pelvis, with six months of luteinizing hormone-releasing hormone (LHRH) agonist. Serum PSA level dropped to 0.01 ng/mL in April 2018 (Figure 1).

At one year of follow-up, serum PSA levels witnessed incremental ascension suggestive of biochemical recurrence of his PCa. A repeated PET/CT C-11 choline, previously considered a gold standard for imaging metastatic prostate adenocarcinoma, did not show any hyper-fixation lesions. A 68-GaPET/CT (PSMA) was performed, which did not show any potential relapse. Therefore, we advocated for serum PSA level surveillance every three months.

**Figure 2:**
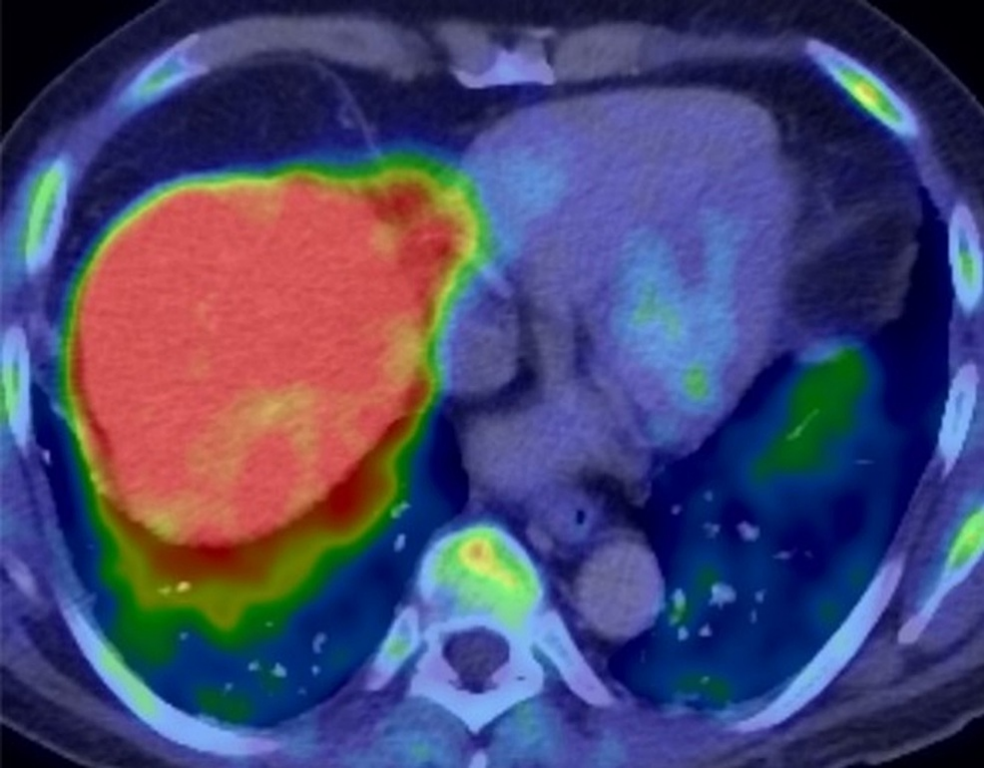
A follow-up PET/CT carbon-11-labeled choline repeated at three-month interval, when prostate-specific antigen measured 1.97 ng/mL, showing no evidence of distant metastasis. The cross-section shown here of the lungs did not reveal any increase in metabolic activity within the lower lobes.

Six months later, serum PSA level reached 1.21 ng/mL. A choline PET/CT scan and magnetic resonance imaging (MRI) of the pelvis were performed, which did not demonstrate any obvious metastatic lesion or local recurrence. The same imaging tests were repeated three months after, when the PSA became 1.97 ng/mL, and again there was no evidence of local recurrence or distant metastasis (Figure 2).

**Figure 3:**
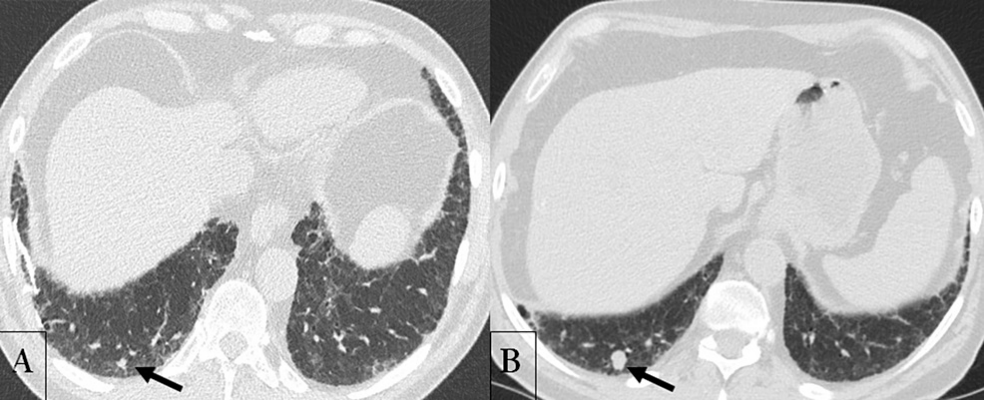
Unenhanced CT scan of the chest performed at one-year interval (A: June 2019; B: July 2020), showing the increase in size of a solitary lung nodule (arrow) located within the posterobasal segment of the right lung, from 4 mm to 15 mm.

At this time, the annual computed tomography (CT) scan of the chest for the patient’s lung fibrosis was scheduled. It showed a more prominent 15-mm nodule in the posterobasal segment of the right lung, increasing in size from 4 mm, on a previous CT of the chest performed a year ago (Figure 3).

A primary lung cancer had been suspected. Fluorodeoxyglucose 18 (FDG)-PET/CT scan and a brain MRI did not reveal any distant metastasis, apart from the abnormal uptake of the solitary lung lesion, exhibiting a SUVmax of 2.9, highly indicative of a primary lung malignancy. As such, the patient received the preliminary diagnosis of a primary lung cancer, alongside a biochemical recurrence of PCa, with no evidence of distant metastasis, based on previous PET/CT scans. Surgical resection was then advised.

The patient had a stable and within normal pulmonary function test, allowing him to undergo a right lung segmentectomy. Fresh-mount study revealed a nodule of a prostatic origin. The nodule was highly vascularized, consisting of plaques and nodules of tumor cells forming glandular structures. Cells were polarized with large nucleolated nuclei with clear cytoplasm. In immunohistochemistry, tumor cells expressed KL1 but not CK7 or CK20. Androgen receptors and PSA were present, whereas TTF1 and other neuroendocrine markers were absent. Ipsilateral regional lymph node dissection was negative for malignant cells. Post-operative serum PSA dropped to 0.8 ng/mL from 1.97 ng/mL preoperatively. The patient is still followed up with serum PSA level measurements every three months.

## Discussion

The incidence of solitary lung metastasis from PCa is reported to be around 0.8% [12]. Until date, only 28 cases of isolated and solitary pulmonary metastasis from PCa have been reported in the literature. The patient in our report is the first one to have a solitary lung metastasis from PCa, which was initially thought of as a primary lung cancer, secondary to his age, history of smoking, and long-standing history of IPF.

The National Comprehensive Cancer Network guidelines define biochemical recurrence as a rising PSA on two or more subsequent occasions [9]. Patients with biochemical recurrence or new metastatic disease are eligible for the standard line of treatment with surgical or chemical castration [13]. Androgen receptor blockage and/or chemotherapy are at the core of metastatic PCa treatment [14].

Due to the patient’s age, previous smoking history, and underlying lung fibrosis, the lung nodule had been considered, at a glance, a primary lung lesion. Yet, the progressive increment of serum PSA levels was highly suggestive of biochemical recurrence, warranting a much closer follow-up. Nevertheless, the performed imaging tests failed to confirm distant metastasis, leading to a presumptive diagnosis of primary lung cancer in this high-risk patient. The rapid decline of serum PSA level following lung nodule resection confirmed its prostatic origin, alongside its characteristic histopathology.

The benefit of solitary pulmonary metastasis resection, in previously treated patients with radical prostatectomy, has been observed in several similar case reports. Wallis et al. highlighted four cases from the literature with complete biochemical response following a solitary pulmonary nodule resection in patients with PCa who previously received radical prostatectomy [15]. Others cases were reported with up to 12 years of remission without any adjuvant therapy [16-18]. However, data on long-term survival outcomes, in similar cases, are lacking [19].

Substantial investigations are of paramount importance to explore patients with biochemical PCa recurrence with the absence of distant metastasis. Gago et al. described three cases of isolated pulmonary metastasis from PCa. One of these cases showed complete biochemical remission with consistently undetectable level of PSA for approximately four years of follow-up [20]. The third case showed very promising overall survival despite the presence of multiple lung nodules. The standard approach for a metastatic disease is a systemic therapy and not a local therapy. This is justified by the probability of the presence of multiple other disease foci. Long-term disease-free survival is confirmed after resection of solitary pulmonary metastasis in many other malignancies such as breast cancer, renal cell carcinoma, colorectal cancer, and sarcomas. Therefore, similar approach in isolated metastatic PCa might be implemented.

## Conclusions

The incidence of solitary pulmonary metastasis in PCa is extremely rare. As PCa may spread solely to the lungs, in-depth investigations are needed in such cases. The management of this type of oligometastatic lesion is a real therapeutic challenge to urologists, oncologists, and pulmonologists. Metastasis-directed therapy may be a reasonable option in the absence of clinical trials.
